# Relapsing Polychondritis Presenting With Hoarseness and Multisystem Involvement: A Diagnostic and Therapeutic Challenge

**DOI:** 10.7759/cureus.74874

**Published:** 2024-11-30

**Authors:** Shamilka De Silva, Monika De Silva, Pradeep K De Silva

**Affiliations:** 1 Internal Medicine, National Hospital of Sri Lanka, Colombo, LKA; 2 Rheumatology, National Hospital of Sri Lanka, Colombo, LKA; 3 Medicine, National Hospital of Sri Lanka, Colombo, LKA

**Keywords:** autoimmune disorder, cyclophosphamide, mcadam criteria, osteoporosis, prednisolone, relapsing polychondritis, zoledronic acid

## Abstract

Relapsing polychondritis (RP) is a rare autoimmune disorder characterized by recurrent inflammation of cartilaginous tissues throughout the body, particularly the ears, nose, eyes, joints, and the respiratory tract. We present a case of a 68-year-old female without previous comorbidities who presented with gradually progressive hoarseness of voice and inflammatory polyarthritis, nasal and ear involvement eventually leading to the diagnosis of RP with concurrent osteoporosis. The diagnosis was made based on Modified McAdam criteria, highlighting the significance of clinical evaluation in guiding diagnosis and treatment decisions. Treatment with intravenous cyclophosphamide and oral prednisolone resulted in significant improvement in symptoms and inflammatory markers. Osteoporosis was managed with intravenous zoledronic acid. This case underscores the importance of considering RP in the differential diagnosis of patients presenting with hoarseness of voice and multisystem involvement, even in the absence of histological confirmation.

## Introduction

Relapsing polychondritis (RP) is a rare autoimmune disorder characterized by recurrent inflammation of cartilaginous tissues, potentially affecting various organ systems [1]. Its prevalence is approximately 4.5 cases per million, affecting all racial groups. However, RP occurs predominantly in Caucasians and has equal frequency in both sexes. It can occur at any age, with the onset of the disease varying between 20 and 60 years old and with the peak age at onset between 40 and 50 years of age, even though it may occur in any age group [2]. RP is considered a complex disorder targeting cartilaginous structures, with the involvement of both humoral and cell-mediated immune systems. Circulating autoantibodies against collagens II, IX, and XI have been detected in RP patients, suggesting that cartilage-specific autoimmunity may play a crucial role in the pathogenesis of RP [3]. Diagnosis of RP can be challenging due to its heterogeneous presentation and lack of specific diagnostic tests. In over 80% of patients, RP is disclosed by auricular chondritis and polyarthritis, though many organs can be potentially involved. Its onset is often insidious, with acute painful inflammatory crisis followed by spontaneous remission of variable duration. This may render diagnosis very difficult at an early stage, with therapeutic delay and consequent increased risk of permanent or life-threatening sequelae [3]. Diagnosis of RP can be challenging due to its heterogeneous presentation and lack of specific diagnostic tests. The clinical features and course of the disease can vary considerably from patient to patient. Here, we report a case of RP presenting with hoarseness of voice, inflammatory arthritis, and nasal and ear involvements complicated by osteoporosis, and discuss the diagnostic approach and management strategies employed.

## Case presentation

**Figure 1:**
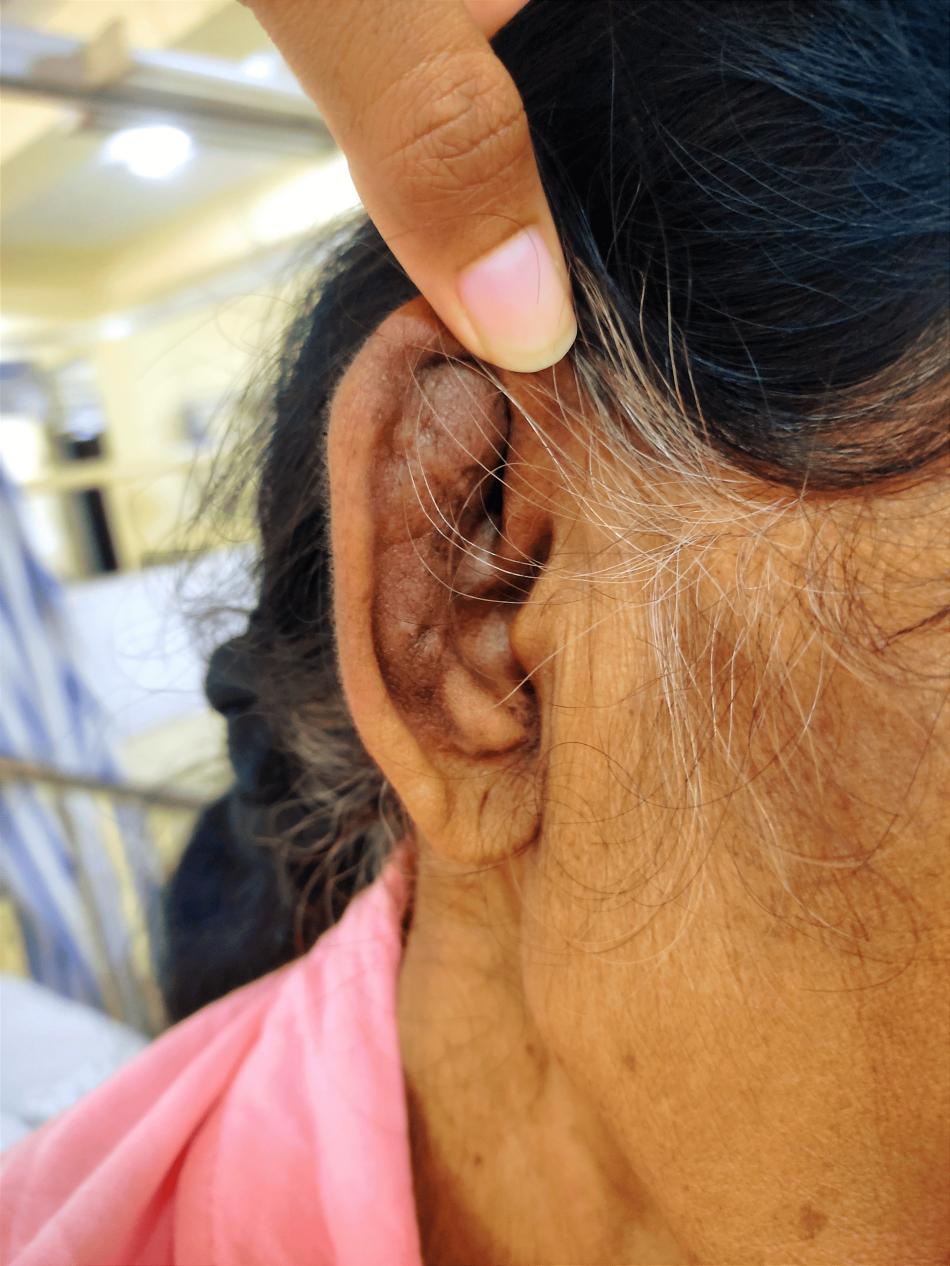
Before treatment with evidence of perichondritis involving cartilage of right ear sparing the lobe

**Figure 2:**
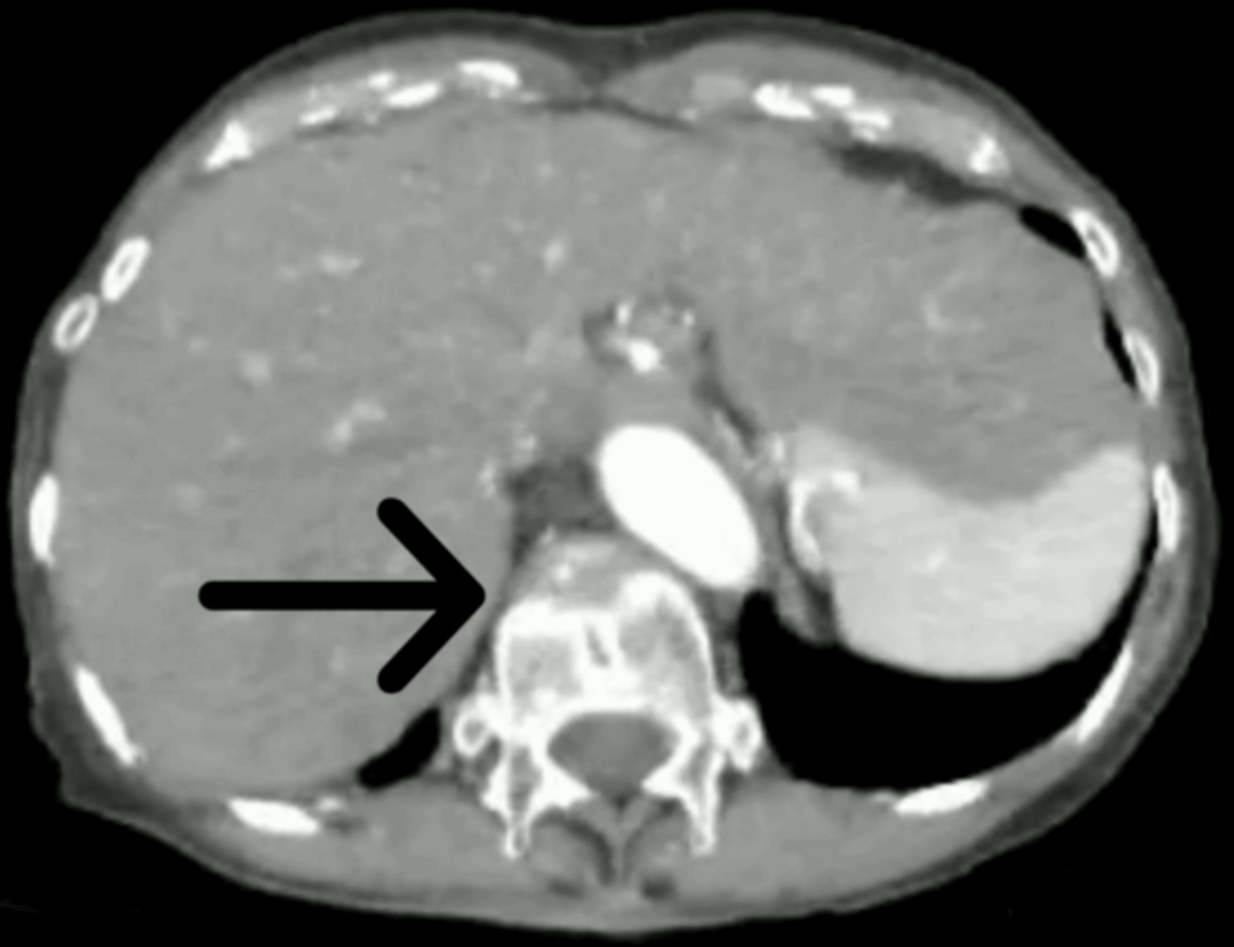
T12 vertebral wedge fracture as indicated by the distortion of vertebral body on axial view CT CT - Computed tomography

**Figure 3:**
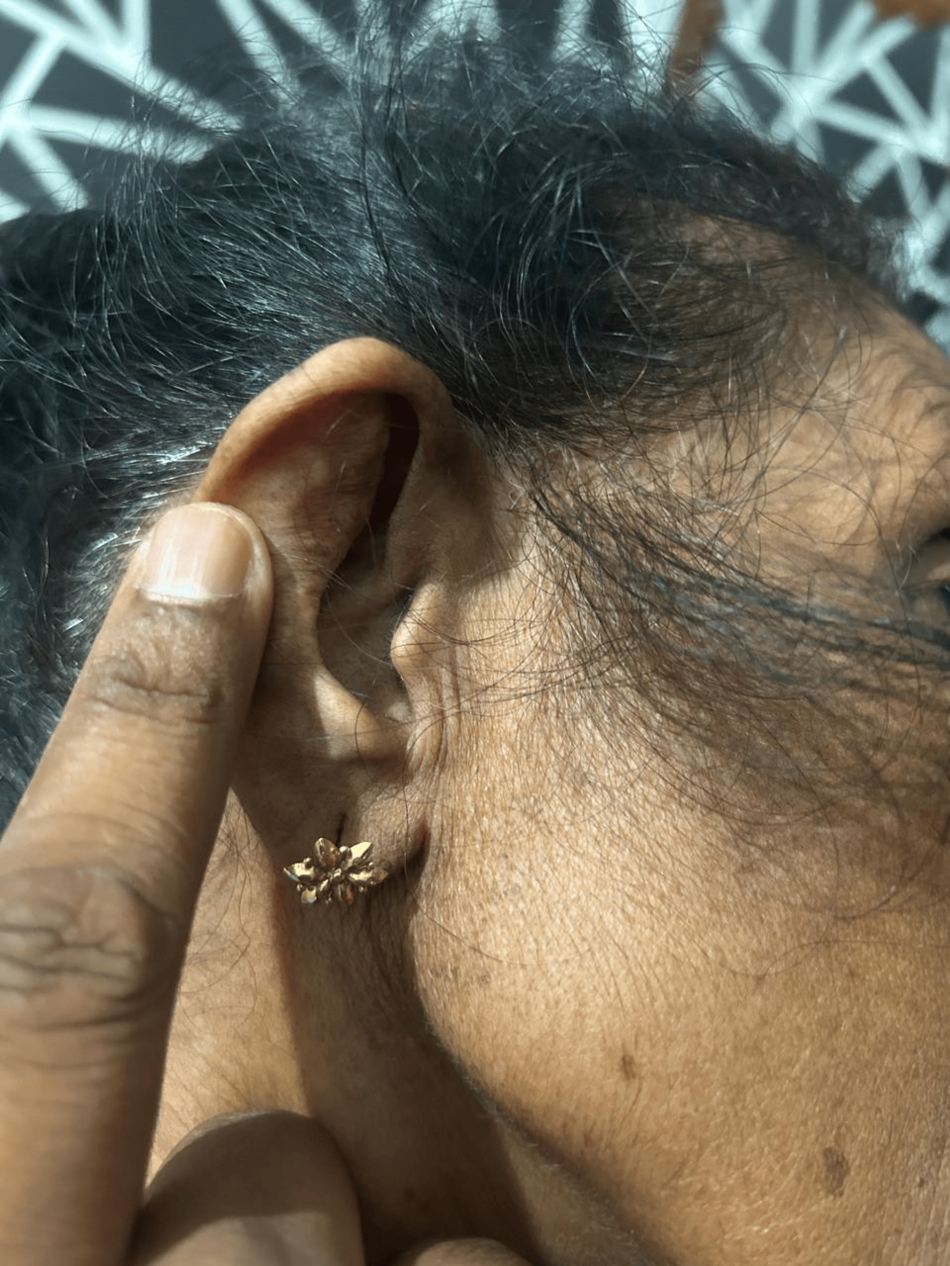
After treatment with evidence of resolving perichondritis of right ear

A 68-year-old female presented with a two-month history of progressively worsening hoarseness of voice. She was initially evaluated by the otolaryngology team. Her thyroid function tests were within the normal range, and fiberoptic laryngoscopy revealed no abnormalities. Her thyroid profile was within range and fiber-optic laryngoscopy was unremarkable. Initial investigations (Table 1) revealed elevated inflammatory markers (ESR 130 mm/hour). Given her Sri Lankan origin, laryngeal tuberculosis was considered a differential diagnosis; however, tuberculosis screening, including tests to rule out associated laryngitis, was negative. Granulomatosis with polyangiitis was also evaluated as a possible cause but both p ANCA and c ANCA were negative. Contrast imaging did not show any local causes for vocal cord compression. The patient was discharged from inward care with subsiding symptoms but re-admitted two months later with worsening hoarseness with fatigue, localized chest pain, and inflammatory arthritis involving large joints and MTP joints in the right foot. She also noticed episodic dryness and irritation in her eyes without visual impairment and she had developed severe pain and swelling of the bridge of the nose. By this time, she had also developed severe pain in her right ear with redness and swelling (Figure 1). On examination, she had bilateral ear swelling with redness which was more on the right side, and a deformed nasal bridge, and her hearing was normal. Although the presenting symptoms were in favor of RP. At the time of her presentation, ophthalmological assessment was unremarkable excluding any scleritis, uveitis, and conjunctivitis and joint examination did not reveal any joint deformities pointing toward an inflammatory polyarthritis. C-reactive protein level was 38mg/dL and positive ANA titer (>1/1,000 nuclear pattern) was noted. Serological tests for retroviral, syphilis, and Brucellosis were negative. Imaging studies focused on her back pain showed no erosive lesions but revealed an old T12 wedge fracture (Figure 2) and evidence of osteoporosis on a DEXA scan (Table 2). Meanwhile, the rheumatoid factor was negative. Fasting blood sugar/HbA1c was within range. Her serum calcium level 2.1 mmol/L and phosphate was 1.10 mmol/L. After treatment, there was evidence of resolving perichondritis of the right ear (Figure 3).

**Table 1:** Summary of investigations ESR - Erythrocyte Sedimentation Rate; CRP - C-reactive Protein; ANA - Anti-nuclear Antibody; TB - Tuberculosis; VDRL - Venereal Disease Research Laboratory Test; ANCA - Anti-nuclear Cytoplasmic Antibody

Investigation	Patient parameter	Reference
White cell count	8.9*10^3^	(4-10) UL
Hemoglobin	11.3	(11-16) g/dL
ESR	130	<20mm 1^st^ hour
CRP	38	<6 mg/L
ANA titer	>1/1,000 – nuclear pattern	
Sputum culture for tuberculosis	Negative	
TB gene xpert	Negative	
Rheumatoid factor	5	<20 U/mL
Brucella antibody	IgG/IgM negative	
VDRL	Negative	
Fasting blood sugar	86	<126 mg/dL
HbA_1_C	5.0%	<6.5%
Serum ionized calcium	2.1	(2.0 – 2.8) mmol/L
Serum phosphate	1.1	(1.0 – 2.8)
ANCA	Negative	

**Table 2:** DXA scan result summary BMC - Bone Mineral Content; BMD - Bone Mineral Density; DXA - Dual-energy x-ray absorptiometry

Region	Area (cm²)	BMC (g)	BMD (g/cm²)	T-score	Z-score	WHO classification	Fracture risk
Right hip (Figure 5)	27.78	16.05	0.578	-2.4	-1.1	Osteopenia	Increased
Lumbar spine L1-L4 (Figure 3)	46.05	25.21	0.547	-4.0	-1.4	Osteoporosis	High
Left hip (Figure 4)	27.82	15.11	0.543	-2.7	-1.4	Osteoporosis	High

## Discussion

The diagnosis of RP was based on Modified McAdam criteria [1], as the patient fulfilled three McAdam criteria: bilateral auricular chondritis, non-erosive seronegative inflammatory arthritis, and nasal chondritis. And she also had respiratory tract chondritis. Histological confirmation was not pursued due to the fulfillment of diagnostic criteria and the patient's clinical response to treatment. Even though the concurrent diagnosis, of osteoporosis is not directly related to RP it further complicated the clinical picture, necessitating comprehensive management as the patient needed to be started on steroids.

Due to the rarity of RP, randomized controlled trials are scarce and there are no evidence-based guidelines for the treatment of RP [3]. The goal of therapy is the control of the inﬂammatory crisis and the long-term suppression of the immune-mediated pathogenetic mechanisms. Non-steroidal anti-inﬂammatory drugs (NSAIDs) may be used for pain control and inflammation in non-severe forms of RP, characterized by involvement of the nose, external ear, or joints only [3,4]. In the presence of organ-threatening disease with RP due to laryngeal involvement activity, she was initiated on intravenous cyclophosphamide (IV CPP) 500 mg weekly for six pulses along with oral prednisolone, resulting in significant improvement in her symptoms (Figure 3) and inflammatory markers. Hoarseness of voice, ear swellings, and nasal bridge pain and deformity showed dramatic improvement. Osteoporosis was managed with intravenous zoledronic acid which is the only available treatment modality in a resource-limited setting. The patient was rapidly tapered off from glucocorticoids due to interference with worsening osteoporosis. Later, she was started on Methotrexate and followed up at an outpatient clinic without having any treatment-related complications.

This case underscores the diagnostic challenges of RP, particularly when the disease initially presents with isolated symptoms. It highlights the importance of considering this rare autoimmune disorder in patients with multisystem involvement. The diagnosis of RP is challenging due to its relapsing nature, the lack of specific diagnostic tests, and the absence of standardized clinicopathological criteria [5]. The diagnosis of RP is made by recognition of a specific pattern of organ involvement, which is based on clinical findings combined with laboratory data and imaging [6]. Careful clinical assessment and investigations should be performed to exclude clinical mimics and other associated rheumatological conditions. The Modified McAdam criteria provided a structured approach to diagnosis, obviating the need for histological confirmation in our case. Biopsy is not necessary when the clinical diagnosis is evident [7]. Biopsy has overall limited utility for the diagnostic approach of RP as there are no pathognomonic features in the histologically, and they vary depending on the stage of the disease. Histologically unremarkable biopsy does not rule out RP [8]. Osteoporosis is likely a separate coexisting pathology given the patient's age, yet it plays a decisive role by influencing the choice of treatment modalities for RP. Therefore, this highlights the need for comprehensive management strategies in patients with RP especially when a patient has other separate concurrent comorbidities that might interfere with the disease or its treatment modalities.

## Conclusions

RP should be considered in the differential diagnosis of patients presenting with chondritis and multisystem involvement, even in the absence of histological confirmation. Continuous evaluation and follow-up of patients presenting with isolated symptoms of RP is of very high importance. The Modified McAdam criteria serve as a valuable tool for guiding diagnosis and treatment decisions in such cases. Early recognition and prompt initiation of tailor-made pharmacological therapy tallying with the patient's comorbidities is crucial in achieving favorable outcomes and preventing both life-threatening and long-term complications.
